# Contribution to the Pathological Physiology of Pneumonia

**Published:** 1853-03

**Authors:** G. Zimmermann


### PATHOLOGY AND PRACTICE OF MEDICINE.

*Contribution to the Pathological Physiology of Pneumonia.* By Dr. G. Zimmermann.—Zimmermann considers the most minute observation of the natural course of acute disease to be the only way of elucidating the laws of these typical processes. Although he thinks those cases most fit for the purpose in which no remedies are employed, yet he does not at all exclude those in which the treatment is active. As an instance of how to observe, he describes the course of a case of pleuro-pneumonia, treated by repeated venesection, application of blisters, and internal administration of antiphlogistic remedies, in moderate doses. From the first day of the fully established disease, till after the return of convalescence, he has noted twice daily (at 8 A. M. as the time of remission, and between 5 and 6 P. M. as the time of exacerbation) the temperature under the tongue, the frequency and other qualities of the pulse, and the number of inspirations on almost every day, the quantity of urine secreted in 24 hours, its specific gravity, and the quantity of lithic acid contained in it. Of great interest are the *critical symptoms*, remarked on the 3d and then again on the 9th day, after which we observe a steady progress to convalescence. The first note on the *temperature* was taken four hours after an attack of general vehement rigors, which the patient himself considered as the commencement of the disease; it was then as high as 10[illegible]°10, which, being 4°14, over the normal warmth of that individual (98°96,) makes Zimmermann inclined to conclude, that the disease had commenced before the occurrence of the rigors. Between the evening of the 2d and the morning of the 3d day, a decrease from 104°0 to 99°87 was observed, coincident with a general abatement of the constitutional symptoms of pyrexia, but on the evening of the 3d day, the temperature was again as high as 105°80 and the physical examination exhibited the signs of hepatization, which had not been present in the morning. Between the evening of the 8th and the morning of the 9th day, the warmth had decreased from 106°16 to 99°50: at the same time all the other febrile symptoms disappeared almost completely, after they had reached a very high degree on the two previous days. It is interesting to remark, that although the frequency of the pulse did not exceed the normal average after that period, yet the temperature increased again to 102°20, and remained so for more than a fortnight longer. The entrance of the change on the 9th day, does not appear to Zimmermann as merely accidental, but he looks at this day as a critical one; and also the coincidence of several alvine evacuations effected by calomel seems important to him, as he thinks that the crisis may be promoted by our remedies, if their action takes place shortly before or on the beginning of a critical day. Concerning the *urine*, the quantity secreted in twenty-four hours was between 9040 and 29,030 grains; the minimum is noted on the 5th, the maximum on the 9th day; the average quantity of 24 hours before the 10th day, was 15,215 grains, with an average specific gravity of 1·0198; between the 10th and the 40th day, 16,510 grains, with 1·0208 specific gravity. The quantitative examination for *urea* was not made before the 8th day, on which 1081 grains were contained in the urine in 24 hours; on the 9th day, only 884 grains; and a few days later, not more than 400 grains (which is about the average quantity during health—*Lehmann*, Physiol. Chem., vol. i. p. 167.) The quantity of *uric acid* was almost normal during the commencement of the disease, but after the sixth day it appeared considerably increased, and reached the maximum on the 9th day, when it amounted to 3770 grains in twenty-four hours (which is at least three times more than the average during health—*Lehmann*, l. c. p. 217.) Zimmermann considers this increase of the uric acid and its salts, at the period of the critical change, as important for the doctrine of *crisis*, as he had opportunity to observe the same phenomenon, not only in pneumonia, but also in typhoid fever, ague, measles, &c. From three examinations of the blood obtained during the first seven days in this case, and from his previous experience, Zimmermann considers as the most important changes in the *inflammatory blood*—that it is less coagulable than the blood in health; that the quantity of fibrine is augmented; that the colored blood-globules are diminished in number, and possess an abnormal disposition for the formation of rolls; that the colorless globules are found in an increased proportion, and snow, likewise a tendency to join in groups.—*Brit. and For. Medico-Chirurg. Rev.*, from *Prager Viertiljahrschrift.*

